# Self-Perceived Halitosis and Related Factors Among the Mask-Wearing Population During the COVID-19 Pandemic in Delhi, India: A Cross-Sectional Study

**DOI:** 10.7759/cureus.32507

**Published:** 2022-12-14

**Authors:** Sonal Bhatia, Vikrant Mohanty, Aswini Y Balappanavar, Kavita Rijhwani, Puneet Chahar, Radhika Gupta

**Affiliations:** 1 Public Health Dentistry, Maulana Azad Institute of Dental Sciences, New Delhi, IND

**Keywords:** covid-19 retro, covid-19, epidemiology, mouth mask, halitosis

## Abstract

Introduction

Halitosis (oral malodor) is a common health condition throughout the world. In India, data on self-reported halitosis and related factors is limited. Mouth mask usage has been made compulsory after the coronavirus disease 2019 (COVID-19) pandemic. This could possibly alter oral microflora and environment and contribute to halitosis. The aim of the study was to determine the prevalence of self-perceived halitosis (SPH) among mask-wearing patients visiting a tertiary care dental hospital in Delhi, India.

Methods

A cross‑sectional study was conducted among a convenience sample of 300 patients visiting a tertiary care dental hospital in the capital of India. SPH status was measured using a self-designed and structured questionnaire containing socio-demographic factors, mask-related habits, and self-perceived oral health status. Statistical analysis was done using Jamovi software (The jamovi project, Sydney, Australia) version 1.8. Descriptive analysis followed by a chi-square test and a multivariate logistic regression test was applied.

Results

Bad breath was perceived by 86 study subjects. Of the participants, 16.7% felt that they had bad breath before the pandemic, and 38% of the participants had an increased perception or feeling of bad breath since regular mask usage. Of the participants, 42.7% felt that they had an increased feeling of dryness in the mouth post-pandemic. SPH status was associated with mask usage frequency (p<0.001), change (p<0.001) and type of mask (p=0.004), increased feeling of dryness (p<0.001), frequency of toothbrushing (p<0.001), self-reported oral disease (p=0.007), and dental treatment in the past 12 months (p=0.005).

Conclusion

The SPH status of the study population was associated with mask-related habits and self-reported oral health status. The findings highlight the importance of possible amendments in preventive and curative care for patients with halitosis post-COVID-19 pandemic.

## Introduction

Halitosis is the presence of unpleasant odor in the exhaled breath. It is a widely prevalent health condition throughout the world, and its prevalence has been estimated at around 31.8% [1] for the general population but can vary up to 65% for certain population groups [2]. It is possible that there is an underestimation of this condition due to the fact that people usually fail to notice their own breath leading to failure of diagnosis [3]. Halitosis can have major detrimental social implications for the sufferer and significantly impact on normal social interactions [4]. The etiology of halitosis is multifactorial; it tends to be both physiological and pathological and might be related with intra- or extra-oral factors. Most instances of halitosis originate intra-orally (called as oral malodor or fetor oris) and are related to factors such as saliva, poor oral hygiene, plaque-related gingival and periodontal diseases, tongue coating, and dental caries [5]. Extra-oral causes of halitosis include diabetes, liver diseases, gastrointestinal (GI) tract ailments, upper and lower respiratory tract diseases, usage of certain medications and foods, alcohol consumption, and smoking. Halitosis can be classified as genuine halitosis (physiological and pathological), pseudohalitosis (complaint of bad breath without its actual existence), and halitophobia (fear of bad breath) [6].

Investigators and clinicians have tried to measure the extent of halitosis with various methods, such as the measurement of volatile sulfur compounds (VSCs) and organoleptic rating, which is considered as the gold standard [7]. Both organoleptic rating and self-perceived halitosis (SPH) are subjective assessments, while the measurement of VSCs is an objective measurement. However, the latter measurement only detects certain odorants exhaled from the mouth. SPH deliberates the presence of the condition, which can be later supplemented with objective measurements. Self-perception of halitosis is a more feasible method, which reduces both cost and time for the examination. Hence, it is a suitable method for epidemiological surveys [1]. Odor perception by an individual is an important criterion for such an assessment. There are numerous factors for odor perception, identification, and threshold with a wide array of interindividual and intraindividual variations including self-rating of olfactory ability, magnitude of exposure, subject bias, and illnesses [8]. Adaptation to one’s own bad breath due to continuous exposure is called as habituation and can result in the underestimation of the condition, although this is arguable [3]. A dilemma exists in relation to this measurement known as the “bad breath paradox,” which is the inability to correctly judge the presence of oral malodor. This means that one can be unaware despite having the condition, and on the other hand, even without having bad breath, one can perceive it, owing to some psychological factors. Hence, this measurement of halitosis is a subjective and complex method [9].

Due to the advent of the severe acute respiratory syndrome caused by coronavirus disease 2019 (COVID-19), there have been various infection control strategies and measures to limit its spread [10]. The direct mode of transmission of the infection is through aerosols formed via surgical procedures and/or in the form of respiratory droplet nuclei. The droplets can spread from person to person via coughing, sneezing, or talking [11]. Hence, respiratory protective devices have been recommended in several communities across the globe to contain the chain of transmission. Various types of respiratory protective devices or “face masks” are used such as (i) disposable N95 and equivalent respirators that achieve efficient filtration of small airborne particles, (ii) disposable surgical masks that are “loose-fitting and disposable device that creates a physical barrier between the mouth and nose of the wearer and potential contaminants in the immediate environment,” and (iii) cloth masks that are nonmedical face coverings, which vary in the number of layers and fit [12]. Prolonged mask-wearing can result in headaches, rashes, acne, rise in temperature, increased breathing resistance, trapped moisture, alteration of respiratory physiology, fatigue, and other psychological effects [13]. Such changes could possibly alter the oral microflora [14] and can result in both intra- and extra-oral halitosis [15].

There has been previous work on self-perceived halitosis and continuous mask-wearing in Brazil and Germany, which showed a positive association between the two factors [16,17]. Nevertheless, there is still predominant and continuous mask usage throughout the world as this pandemic has no foreseeable end. It is possible that continuous mask-wearing may influence the concentrations of odorants inside the mask or the psychology of an individual giving rise to an increased perception of bad breath. Therefore, we hypothesized that this practice could change self-perceived halitosis (SPH) among individuals. The aim of the present study was to determine the prevalence of self-perceived halitosis and its associated factors among mask-wearing patients visiting a tertiary care dental hospital in Delhi, India.

## Materials and methods

Sample and study design

The present cross-sectional study was carried out among patients visiting a public-sector, tertiary care dental hospital in Delhi, India, from June to August 2021. A protocol was developed prior to the commencement of the study by three authors (SB, AYB, and VM).

**Figure 1:**
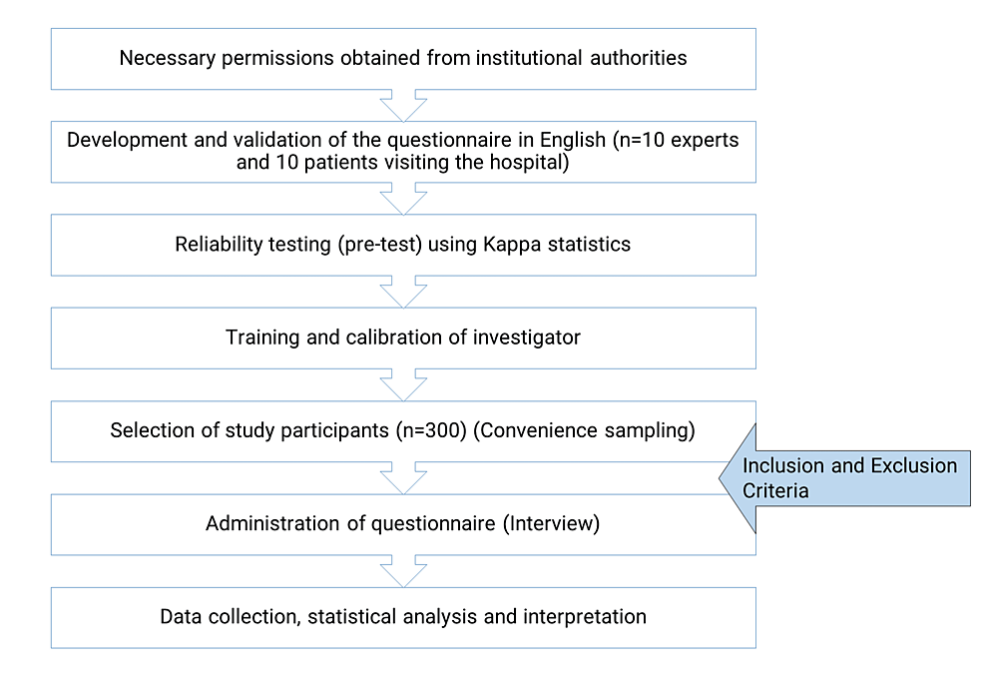
Flow of the study

A single population proportion formula was used to estimate the sample size by using statistical software Epi Info (Centers for Disease Control and Prevention, Atlanta, GA) (version 3.5.4) by considering the Z-score at 95% confidence interval (CI) of 1.96, the absolute precision of 5%, and the prevalence of self-perceived halitosis of 22.8% [18]. The required sample size was calculated as 271, which was rounded off to 300 to account for the assumption of nonresponders (10%). The inclusion criteria were participants that gave their informed consent to the study and were able to comprehend the English language. The exclusion criteria were patients who were being referred to the hospital for emergency services. The sample was selected by convenience sampling technique from the outpatient clinic of the hospital. The flow of the study is depicted in Figure 1.

Questionnaire items and data collection

A self-designed, structured, and interviewer-administered questionnaire was developed in English language by two authors (SB and AYB). Questionnaire items were formulated according to a previous study and were adapted according to the population set [18,19]. The questionnaire was divided into five subsections: socio-demographic details, mask-related habits, self-perceived halitosis status, risk factors, and self-perceived oral health status. The questionnaire comprised 23 closed-ended questions including Likert scale for the assessment of SPH status.

The questionnaire was subsequently validated for face and content validity by groups of target population and experts (each 10 in number) (scale-content validity index {S-CVI}=0.81). A pre-test reliability testing (kappa statistics and Cronbach’s α=0.78) was further done followed by training and calibration of the interview personnel (SB and RG). A set of questions were assessed by other reviewers (VM, AYB, KR, and PC) for comprehension, semantics, and clarity. Questions were rephrased until they were determined to be appropriate and understandable. Any discrepancies found were discussed with the author (AYB), and consensus was reached. The final questionnaire can be found in Appendices.

All participants were explained the purpose of the study and gave an informed consent prior to the commencement of the study. Participants were interviewed in a closed room in the hospital, and the interview lasted for an average of 20-30 minutes for each subject. Socio-demographic details such as age, gender and occupation, and income and education were recorded prior to the questionnaire. Further, the socio-economic status of the individual was calculated according to the modified Kuppuswamy scale (2021) [20]. Interviewees answered questions on mask-wearing frequency and related habits, systemic conditions, oral health parameters, oral hygiene habits, feeling of dryness after mask usage, bad breath status before and after the pandemic, and other bad breath-related concerns.

Statistical analysis

The data collected from the questionnaire was entered in a digital spreadsheet (MS Excel®, Microsoft® Corp., Redmond, WA) with proper coding and was analyzed statistically using Jamovi statistical software program (The jamovi project, Sydney, Australia) (version 1.8). Descriptive data was obtained as frequency (%), mean±standard deviation (SD), interquartile range, and median. The inferential statistics (using chi-square test) was obtained after checking for normal distribution of the data using Kolmogorov-Smirnov test. The level of significance was p<0.05. These were further analyzed by multivariate logistic regression analysis (at confidence level of 95%) to determine variables associated with the presence/absence of SPH. All significantly associated variables were selected for the full initial logistic regression model. Subsequently, variables were manually removed from this initial model, and changes in coefficients were observed. Variables were retained in the final logistic models if found to be significant (p<0.05). Results obtained were tabulated and analyzed by the primary author (SB) with the help of other authors (AYB, PC, and KR).

The reporting of the study was in accordance with the Strengthening the Reporting of Observational Studies in Epidemiology (STROBE) reporting guideline for cross-sectional studies [21].

Ethical considerations

The present cross-sectional study was conducted in accordance with the principles embodied in the Declaration of Helsinki (seventh revision, 2013) and in accordance with local statutory requirements. All participants gave written informed consent following an explanation of the research objectives. Hospital administrations were informed of the research goals, and their permission was obtained prior to starting the study. The collected data was summarized and reported in the aggregate and used only for scientific purposes. Participants were informed about the purpose of the study, the data protection rights, and the right to refuse participation in the study or to terminate the participation without reasoning or penalty. Survey methodology was applied with minimal risk or harm to study participants.

## Results

The sample consisted of 154 males (51.3%) and 146 females (48.7%). The majority of the samples were in the age group of 16-29 years (n=166, 55.3%). Around 53% (n=159) belonged to the upper-middle socio-economic class. Most of the study participants wore masks often (n=103, 34.3%) and always (n=99, 33%). Cloth mask (n=144, 48%) was commonly used and reportedly worn majorly for 2-4 hours (n=86, 28.7%). The majority (n=211, 70.1%) of the participants felt that masks should be frequently worn.

**Figure 2:**
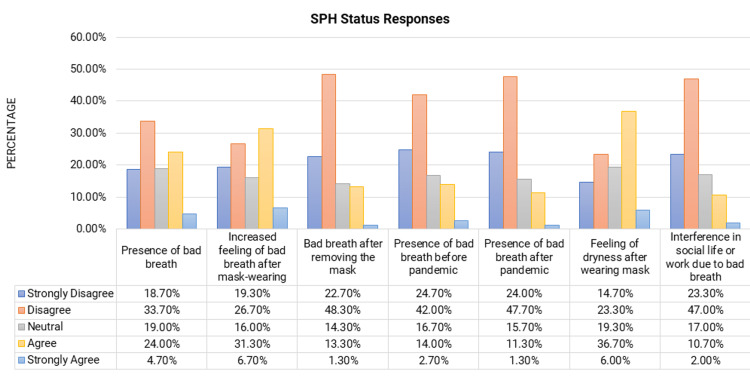
Percentage distribution of responses to self-perceived halitosis status by the study subjects SPH: self-perceived halitosis

Figure 2 shows the responses to self-perceived halitosis status part of the questionnaire. The SPH status was determined by the question, “Do you think you have bad breath?” The responses to this question were dichotomized into “yes” and “no” for better interpretability, “yes” being those participants who strongly agreed and agreed to the statement and “no” being those who were neutral, disagreed, or strongly disagreed. Overall, SPH was prevalent (“yes”) in 28.66% (n=86). Of the participants, 16.7% (n=50) felt that they had bad breath before the COVID-19 pandemic, and 38% (n=114) of the participants had an increased perception or feeling of bad breath since regular mask usage. Of the participants, 42.7% (n=128) felt that they had increased feeling of dryness in the mouth post-pandemic.

Table 1 shows the association of SPH status with socio-demographic factors. Demographic details such as gender, age, and socio-economic status were not significantly associated with SPH status among the study subjects.

**Table 1:** Association of self-perceived halitosis (SPH) with socio-demographic factors *P value<0.05: significant df: degree of freedom; P value: probability value

Study participant characteristics	Whole sample (N=300)	SPH, yes (N=86)	SPH, no (N=214)	Chi-square value	df	P value
	N (percentage within the column)	N (percentage within the row)				
Gender						
Male	154 (51.3)	40 (26.0)	114 (74.0)	1.12	3	0.289
Female	146 (48.7)	46 (31.5)	100 (68.5)			
Age (in years)						
16-29	166 (55.3)	47 (28.3)	119 (71.7)	0.732	4	0.947
30-39	50 (16.7)	14 (28)	36 (72)			
40-49	34 (11.3)	9 (26.5)	25 (73.5)			
50-59	37 (12.3)	11 (29.7)	26 (70.3)			
≥60	13 (4.3)	5 (38.5)	8 (61.5)			
Socio-economic status						
Upper	67 (22.3)	17 (25.4)	50 (74.6)	1.30	4	0.728
Upper middle	159 (53)	15 (31.4)	109 (68.6)			
Lower middle	44 (14.7)	11 (12.8)	33 (75.0)			
Upper lower	30 (10)	8 (26.7)	22 (73.3)			
Lower	0 (0)	0 (0)	0 (0)			

Table 2 shows the association of mask-related habits with SPH status. Mask-wearing frequency was found to be significantly associated with having SPH (p<0.0001) wherein 84.5% of the study subjects who often wore mask had SPH while 58.3% who wore mask rarely did not have SPH. Of the subjects who used surgical mask, 78.2% did not agree to having bad breath, while one-third of the subjects who wore a cloth mask agreed to having bad breath (p=0.004). The daily mask-wearing period was significantly associated with SPH (p=0.034) wherein 100% of the individuals who wore mask for long hours (>10 hours/day) did not perceive halitosis. Moreover, a significant proportion of people who were changing their mask once (81%) or twice (77.4%) a day did not have SPH (p<0.001). The time period of mask disposal was significantly associated with SPH (p=0.022).

**Table 2:** Association of self-perceived halitosis (SPH) with mask-related habits *P value<0.05: significant df: degree of freedom; P value: probability value

Study participant characteristics	Whole sample (N=300)	SPH, yes (N=86)	SPH, no (N=214)	Chi-square value	df	P value
	N (percentage within the column)	N (percentage within the row)				
Do you wear a mask?						
Rarely	24 (8)	10 (41.7)	14 (58.3)	42.09	3	<0.0001*
Sometimes	74 (24.7)	34 (46.0)	40 (54.0)			
Often	103 (34.3)	87 (84.5)	16 (15.5)			
Always	99 (33)	77 (77.8)	22 (22.2)			
Which of the following masks do you use?						
N95	27 (9)	8 (29.6)	19 (70.4)	17.4	5	0.004*
Surgical mask	55 (18.3)	12 (21.8)	43 (78.2)			
Cloth mask	144 (48)	48 (33.3)	96(66.7)			
Home-made mask	10 (3.3)	6 (60.0)	4(40.0)			
Others	2 (0.7)	2 (100.0)	0(0)			
Multiple	62 (20.7)	10 (16.1)	52(83.9)			
How many hours/day do you wear a mask?						
0-2 hours	46 (15.3)	14 (30.4)	32 (69.6)	10.4	4	0.034*
2-4 hours	86 (28.7)	26 (30.2)	60 (69.8)			
4-6 hours	80 (26.7)	20 (25.0)	60 (75.0)			
6-10 hours	70 (23.3)	26 (37.1)	44 (62.9)			
>10 hours	18 (6)	0 (0)	18 (100.0)			
How frequently do you change the mask in a day?						
None	110 (36.7)	46 (41.9)	64 (58.1)	15.0	2	<0.001*
1 time	84 (28)	16 (19.0)	68 (81.0)			
2 times	106 (35.3)	24 (22.6)	82 (77.4)			
Do you think masks should be frequently worn?						
Strongly disagree	10 (3.3)	6 (60.0)	4 (40.0)	9.03	4	0.060
Disagree	22 (7.3)	10 (45.5)	12 (54.5)			
Neutral	57 (19)	16 (18.6)	41 (19.2)			
Agree	151 (50.3)	40 (28.1)	111 (71.9)			
Strongly agree	60 (20)	14 (23.3)	46 (76.7)			
How do you maintain the cleanliness of the mask?						
Dispose of after use	71 (23.7)	18 (25.4)	53 (74.6)	3.47	4	0.482
Wash it with soap and water	185 (61.7)	58 (31.4)	127 (68.6)			
Sanitize it	18 (6)	4 (22.2)	14 (77.8)			
Don’t clean it	4 (1.3)	2 (50.0)	2 (50.0)			
Any other	22 (7.3)	4 (18.2)	18 (81.8)			
After how many days do you dispose of/wash your mask?						
0-2 days	183 (61)	46 (25.1)	137 (74.9)	11.5	4	0.022*
2-4 days	75 (25)	20 (26.7)	55 (73.3)			
4-6 days	22 (7.3)	12 (54.5)	10 (45.5)			
6-10 days	2 (0.7)	0 (0.0)	2 (100.0)			
>10 days	18 (6)	8 (44.4)	10 (55.6)			

Table 3 shows the association of risk factors and oral health status with SPH status of the subjects. SPH was reported by all individuals with respiratory diseases (p=0.033). Out of the individuals who consumed both tea and coffee, 37.7% reported having bad breath (p=0.019). The reporting of bad breath was significantly associated (p=0.007) with having gum disease (46.7%), dryness (33.3%), or multiple oral conditions (50%). There was found to be a strong association between frequency of brushing and SPH (p<0.001) wherein 82.9% of the individuals who brushed twice daily did not report bad breath. Most of the individuals who had scaling (71.4%), filling (83.3%), and extraction (86.2%) done in the past 12 months did not have SPH, while most of those who had root canal treatment done (62.5%) had SPH (p=0.005).

**Table 3:** Association of self-perceived halitosis (SPH) with risk factors and oral health status *P value<0.05: significant df: degree of freedom; P value: probability value; GI: gastrointestinal

Study participant characteristics	Whole sample (N=300)	SPH, yes (N=86)	SPH, no (N=214)	Chi-square value	df	P value
	N (percentage within the column)	N (percentage within the row)				
Do you have any medical condition?						
No	262 (87.3)	76 (29.0)	186 (71.0)	0.118	1	0.732
Yes	38 (12.7)	10 (26.3)	28 (73.7)			
Do you have any of the following condition(s)?						
None	262 (87.3)	74 (28.2)	188 (71.7)	12.1	5	0.033*
Diabetes mellitus	8 (2.6)	0 (0)	8 (100.0)			
Respiratory disorders	4 (1.3)	4 (100.0)	0 (0)			
Chronic acid reflux and GI disorders	4 (1.3)	2 (50.0)	2 (50.0)			
Others	16 (5.3)	6 (37.5)	10 (62.5)			
Multiple	6 (2)	0 (0)	6 (100.0)			
Do you consume/smoke/chew tobacco in any form?						
None	250 (83.3)	72 (28.8)	178 (71.2)	1.27	3	0.735
Smoke form	36 (12)	10 (27.8)	26 (72.2)			
Smokeless form	10 (3.3)	2 (20.0)	8 (80.0)			
Dual	4 (1.3)	2 (50.0)	2 (50.0)			
Do you consume any of the following?						
None	39 (13)	6 (15.4)	33 (84.6)	9.95	3	0.019*
Tea	127 (42.3)	30 (23.6)	97 (76.4)			
Coffee	12 (4)	4 (33.3)	8 (66.7)			
Both	122 (40.7)	46 (37.7)	76 (62.3)			
Do you consume alcohol?						
No	252 (84)	76 (30.2)	176 (69.8)	1.71	1	0.19
Yes	48 (16)	10 (20.8)	38 (79.2)			
Do you have any of the following oral conditions?						
None	82 (27.3)	22 (26.8)	60 (73.2)	17.7	6	0.007*
Decay	116 (38.7)	22 (19.0)	94 (81.0)			
Gum disease	30 (10)	14 (46.7)	16 (53.3)			
Pyorrhea	16 (5.3)	4 (25.0)	12 (75.0)			
Dryness of mouth	12 (4)	4 (33.3)	8 (66.7)			
Others	12 (4)	4 (33.3)	8 (66.7)			
Multiple	32 (10.7)	16 (50.0)	16 (50.0)			
Which of the following oral hygiene aids do you use?						
Toothpaste and toothbrush	281 (93.6)	78 (27.8)	203 (72.2)	1.86	2	0.601
Mouthwash	135 (45)	44 (32.6)	91 (67.4)			
Interdental aids	10 (3.3)	2 (20.0)	8 (80.0)			
How frequently do you brush your teeth?						
None	8 (2.7)	4 (50.0)	4 (50.0)	17.5	3	<0.001*
Once/day	143 (47.7)	50 (35.0)	93 (65.0)			
Twice/day	129 (43)	22 (17.1)	107 (82.9)			
>Two times	20 (6.7)	10 (33.3)	10 (66.7)			
Have you undergone any oral/dental procedure in the past 12 months?						
None	145 (48.3)	48 (33.1)	97 (66.9)	18.6	6	0.005*
Scaling	42 (14)	12 (28.6)	30 (71.4)			
Filling	48 (16)	8 (16.7)	40 (83.3)			
Root canal treatment	16 (5.3)	10 (62.5)	6 (37.5)			
Extraction	29 (9.7)	4 (13.7)	25 (86.2)			
Any other	4 (1.3)	0 (0)	4 (100.0)			
Multiple	16 (5.3)	4 (25.0)	12 (75.0)			

The final multivariate logistic model for the SPH status with retained variables (those found significant) is shown in Table 4. For mask-related habits, SPH was significantly associated with often (odds ratio {OR}=7.61) and always (OR=4.90) mask-wearing, change of mask once (OR=0.33) and twice (OR=0.4) per day, and mask disposal/washing after 4-6 days (OR=3.57). For risk factors, the only retained variable was both tea and coffee consumption (OR=3.33). For oral health status, gum disease (OR=2.39), multiple oral conditions (OR=2.73), and twice daily brushing (OR=0.21) were significant. Further, dental treatment status for the past 12 months was significant for fillings (OR=0.4), root canal treatment (OR=3.37), and extractions (OR=0.32).

**Table 4:** Final adjusted multivariate logistic regression analysis to assess the association of different determinants with self-perceived halitosis (SPH) status P value: probability value; OR: odds ratio; CI: confidence interval

Independent variables	Regression analysis with SPH (yes - no)		
Selected variable - reference variable	P value	OR	CI
Mask-related habits			
Do you wear a mask?			
Often - rarely	<0.001	7.61	2.88-20.1
Always - rarely	<0.001	4.90	1.91-12.54
How frequently do you change the mask in a day?			
1 time - none	<0.001	0.33	0.17-0.64
2 times - none	0.003	0.40	0.22-0.74
After how many days do you dispose of/wash your mask?			
4-6 days - 0-2 days	0.006	3.57	1.45-8.82
Risk factors			
Do you consume any of the following?			
Tea and coffee - none	0.012	3.33	1.3-8.55
Oral health status			
Do you have any of the following oral conditions?			
Gum disease - none	0.049	2.39	1.0-5.68
Multiple - none	0.020	2.73	1.17-6.37
How frequently do you brush your teeth?			
Twice/day - none	0.034	0.21	0.05-0.88
Have you undergone any oral/dental procedure in the past 12 months?			
Filling - none	0.033	0.40	0.18-0.93
Root canal treatment - none	0.026	3.37	1.16-9.81
Extraction - none	0.046	0.32	0.11-0.98

## Discussion

The present study showed that 28.66% of the sample population self-reported to have bad breath. The literature reveals that the prevalence of SPH is variable, e.g., 13.3% in Germany [17] and 52.5% in India [22]. The self-perception of odor is influenced by various factors, both physiological and psychological. These judgments are subjective and can vary from person to person. The self-assessment of halitosis is a true patient-centered measurement, since it involves the individual in the diagnosis and makes them aware of it, and hence highly relevant to contemporary medical research [16].

Due to the novel situation of a global pandemic and continuous mask-wearing, people may have become more aware of their breath because a mask creates a physical barrier through which breath is exhaled and physically returned back. Our findings showed that individuals who wore masks often had 7.61 times higher chances of reporting halitosis than those who wore them rarely. In the present study, 38% of the participants agreed to having increased feeling of bad breath since wearing masks. A previous study showed that 33.8% of the study sample changed their self-perceived breath odor after wearing a mask during the pandemic [16]. The self-perception of bad breath plays a key role in understanding other individual and psychological perspectives of halitosis.

Additionally, 29.6% of N95 mask wearers, 21.8% of surgical mask wearers, and 33.3% of cloth mask wearers reported SPH. Contrarily, in a previous study, it was found that surgical mask-wearing impacted on halitosis scores more than N95 mask-wearing [17]. This difference could be due to socio-demographic and cultural characteristics of the observed population. In India, people of lower socio-economic status (linked to poorer health status) might prefer to wear cloth masks over N95 due to the costliness of the latter [23]. It is difficult to draw definite conclusions from these findings because of the fact that the self-perception of breath malodor has complex psychological factors associated with it. There is a concept of “breath odor image,” which correlates with the concept of how one perceives oneself, i.e., “body image,” which is highly personalized and socially influenced subjective experiences relating to perceptions, thoughts, and feelings about the body [9].

In the present study, with the increase in daily wearing time of masks, the reporting of halitosis increased with the exception of those who had the longest wearing time (>10 hours). None of these participants reported halitosis. This is consistent with a previous study wherein changes in perceived halitosis decreased with increased period of mask usage [16]. Contrarily, in another study, SPH increased with increased wearing time [17]. Perhaps, in our study, participants with extreme wearing hours did not report halitosis because of the phenomenon of habituation and adaptation. Habituation, or decreased behavioral response, to odors is created by repeated exposure, resulting in reduced perceptual intensity of odor with time [24].

Participants who changed their masks daily were 67% less likely to have SPH. Furthermore, individuals who disposed their masks later (4-6 days) were 3.57 times more likely to have SPH than those who did it earlier (0-2 days). No previous literature reports such findings. Within this context, it could be hypothesized that proper mask hygiene is a possible protective factor for the prevention of halitosis.

Although not retained in final adjusted logistic regression model, the self-report of any medical condition was significantly associated with halitosis status. Halitosis was reported by 27.8% of the participants with the presence of a medical condition. Furthermore, all participants who reported a respiratory condition reported to have bad breath. The presence of systemic condition has previously been associated with SPH [25]. Various systemic conditions are associated with halitosis alterations. Medications, drug reactions, or side effects can also cause xerostomia and be an indirect cause of oral malodor [5]. Certain medical conditions have been considered causative for halitosis such as diabetic ketoacidosis; respiratory diseases such as sinusitis, tonsillitis, and bronchiectasis; and gastro-esophageal reflux disease [6].

It was observed that 42.7% of individuals reported feeling of dryness since continuous mask usage. Previously, it has been self-reported that dry mouth was associated with SPH [16]. Feeling of dryness or xerostomia predisposes an individual to halitosis [5].

Oral malodor can be a ramification of lifestyle habits, such as the consumption of certain food and beverages, or deleterious habits such as smoking, chewing tobacco, or alcohol consumption [5]. In the present study, individuals consuming both tea and coffee are 3.33 times more likely to have SPH as compared to those consuming no beverages. Evidence linking halitosis to tea and coffee consumption is scarce, although it might be beneficial for oral health owing to their antimicrobial properties [26]. Tobacco smoke contains VSCs, which is a type of malodorant. Tobacco also predisposes to dry mouth, which is a causative factor for halitosis [5]. In this present study, 38.8% of tobacco users had SPH, although the association was not significant. Previously, tobacco use [18] and smoking [25] have been shown to be associated with SPH. Hence, education against these hazardous lifestyle habits is necessary for every medical practitioner.

Our study also assessed the association of the frequency of toothbrushing on halitosis. Individuals who brushed their teeth twice daily were 79% less likely to have SPH as compared to those who did not brush. The role of toothbrushing is already recognized for optimal oral hygiene to prevent and treat halitosis [27]. Moreover, 67.4% of mouthwash users did not report the condition. These oral hygiene practices can result in lower prevalence of SPH [28]. Certain mouthwashes contain components such as chlorhexidine gluconate, cetylpyridinium chloride, and essential oils that can reduce microbial counts and/or neutralize the odor [5,29].

In the study, people who had self-reported gum diseases were 2.39 times more likely to have SPH than those who did not report any oral disease. Similarly, people who reported multiple oral conditions were 2.73 times more likely to report halitosis. This is in agreement with previous studies that have shown a positive association of gingivitis and periodontal disease with halitosis [30]. Gingival and periodontal conditions have been recognized as the main intra-oral causes of halitosis [5]. 

In the past 12 months, participants who had restorations done were 60% less likely to report halitosis, those who had extractions done were 68% less likely to report halitosis, and those who underwent root canal treatment were 3.37 times more likely to report halitosis. There were no previous studies assessing this association. These dental treatment procedures are therapeutic in nature. However, these may also, rather contrarily, signify the presence of poor oral hygiene. Multiple dental procedures are usually done for patients with poor oral health status. Hence, we see bidirectional nature in the regression results, wherein restorations and extractions seem to have a protective effect while root canal procedures are possibly causative. The latter lies at the tertiary level of prevention, done for cases in which dental caries has been neglected at earlier stages, indicating a poorer attitude toward oral health.

It is imperative for health professionals to understand the etiology of halitosis to identify risk factors, diagnose and treat the condition, and hence deliver higher-quality services and health education. The association of continuous mask-wearing and halitosis can be a way forward for future analytical research to better understand the causal links between the two and their correlates. This could help in generating evidence to issue better public health policies and measures regarding proper mask use and precautions for future pandemics.

Limitations

The present cross-sectional study has certain limitations. As the present study was conducted during the COVID-19 pandemic, convenience sampling method was used to acquire the study sample. Due to the nature of the questionnaire study, some response and interviewer bias may have occurred. Certain individuals were ashamed or hesitant to admit that they have bad breath to the investigator leading to social desirability bias. Due to Likert type of questioning, there may also be some end-aversion bias present in the responses. Some questions compelled participants to recall certain medical and dental history and hence may have contributed to some recall bias. The measurement of halitosis used in the study is a subjective type of assessment and may not actually reflect the presence of the condition. Nevertheless, the findings in the study are relevant and can be the basis for future research.

## Conclusions

Self-perceived halitosis status was significantly associated with mask-related habits. The findings support the presence of increased dryness of the mouth upon continuous mask usage, which is a contributing factor for oral malodor. Self-reported oral health status was associated with the reporting of halitosis, including oral hygiene habits, the presence of gingival disease, and dental treatment history. The findings highlight the importance of possible amendments in preventive and curative care for patients with halitosis post-COVID-19 pandemic. Further research is needed to confirm the results of the present study. In addition, there is also a need for future research on mask-wearing and its correlates for oral health to generate comprehensive public health guidelines.

## Appendices

Questionnaire

Consent Form

I, ……………………………, hereby declare that I give consent to participate in the study “Self-perceived halitosis and mask-related habits among patients at a tertiary care hospital in Delhi” conducted by Dr. Sonal Bhatia, postgraduate student from the Department of Public Health Dentistry, Maulana Azad Institute of Dental Sciences, New Delhi. I have been explained the aim and objectives of the study in an appropriate language. I also understand that the data collected from this study will be used for research purposes and presentation. I have been told that my information will be kept confidential.

I also reserve my rights to withdraw myself from the study whenever I wish.

Signature …………………………

Table 5 presents the final questionnaire.

**Table 5:** Questionnaire administered to the study participants OPD: outpatient department

To be filled out by the investigator					
Section I: socio-demographic details					
Date:	Name:	OPD number:	Age/sex:	Phone number:	
Address:	Marital status:	Education status:	Income (monthly):	Occupation:	
Section II: mask-related habits					
Q1. Do you wear a mask?	Never	Rarely	Sometimes	Often	Always
Q2. Which of the following masks do you use?	N95	Surgical mask	Cloth mask	Home-made mask	Others
Q3. How many hours/day do you wear a mask?	0-2 hours	2-4 hours	4-6 hours	6-10 hours	>10 hours
Q4. How frequently do you change the mask in a day?	None	1 time	2 times	3 times	>3 times
Q5. Do you think mask should be frequently worn?	Strongly disagree	Disagree	Neutral	Agree	Strongly agree
Q6. How do you maintain the cleanliness of the mask?	Dispose it off after use	Wash it with soap and water	Sanitize it	Don’t clean it	
Q7. After how many days do you dispose of/wash your mask?	0-2 days	2-4 days	4-6 days	6-10 days	>10 days
Section III: self-perceived halitosis status					
Q8. Do you think you have bad breath?	Strongly disagree	Disagree	Neutral	Agree	Strongly agree
Q9. Since wearing the mask, have you experienced increased feeling of bad breath?	Strongly disagree	Disagree	Neutral	Agree	Strongly agree
Q10. Do you feel you have bad breath even after removing the mask?	Strongly disagree	Disagree	Neutral	Agree	Strongly agree
Q11. Did you feel that you had bad breath before the COVID-19 pandemic?	Strongly disagree	Disagree	Neutral	Agree	Strongly agree
Q12. Has your bad breath interfered with your social life or workplace?	Never	Rarely	Sometimes	Often	Always
Q13. Has anyone told you that your mouth smells since COVID-19?	Never	Rarely	Sometimes	Often	Always
Q14. Do you feel an increase in dryness of the mouth after mask-wearing?	Strongly disagree	Disagree	Neutral	Agree	Strongly agree
Section IV: risk factors					
Q15. Do you have any of the following conditions (multiple answers)?	Diabetes mellitus	Respiratory diseases (tuberculosis, sinusitis, tonsillitis, etc.)	Liver diseases (hepatitis and liver failure)	Chronic acid reflux or any other GIT disorder	Others
Q16. Do you consume/smoke/chew tobacco in any form?	None	Smoke form	Smokeless form	Dual	-
Q17. How many cigarette/bidis/dips/chew do you smoke/consume per day?	10 or less	11-20	21-30	30 or more	-
Q18. Do you consume any of the following?	Tea	Coffee	Both	-	-
Q19. How frequently do you consume alcohol?	Never	Occasional use	Once/day	>Once/day	-
Section V: self-perceived oral health status					
Q20. Do you have any of the following oral conditions (multiple answers)?	Decay	Gum disease	Pyorrhea	Dryness of the mouth	Others
Q21. Which of the following oral hygiene aids do you use (multiple answers)?	Toothpaste and toothbrush	Mouthwash	Interdental aids	Others	
Q22. How frequently do you brush your teeth?	None	Once/day	Twice/day	>2 times	
Q23. Have you undergone any oral/dental procedure in the past 12 months (multiple answers)?	Scaling	Filling	Root canal treatment	Extraction	Others
